# Imaging and Management of Bladder Cancer

**DOI:** 10.3390/cancers13061396

**Published:** 2021-03-19

**Authors:** Vincenzo K. Wong, Dhakshinamoorthy Ganeshan, Corey T. Jensen, Catherine E. Devine

**Affiliations:** Department of Abdominal Imaging, The University of Texas MD Anderson Cancer Center, 1515 Holcombe Blvd, Houston, TX 77030, USA; dganeshan@mdanderson.org (D.G.); cjensen@mdanderson.org (C.T.J.); catherine.devine@mdanderson.org (C.E.D.)

**Keywords:** bladder cancer, urothelial carcinoma, imaging, management, CT, MRI, urography

## Abstract

**Simple Summary:**

Bladder cancer is a complex disease, the sixth most common cancer, and one of the most expensive cancers to treat. In the last few decades, there has been a significant decrease in the bladder cancer-related mortality rate, potentially related to decreased smoking prevalence, improvements in diagnosing bladder cancer, and advances in treatment. Those advances in diagnostic tools and therapies and greater understanding of the disease are helping to evolve how bladder cancer is managed. The purpose of this article is to provide a review of bladder cancer pathology, diagnosis, staging, radiologic imaging, and management, and highlight recent developments and research.

**Abstract:**

Methods: Keyword searches of Medline, PubMed, and the Cochrane Library for manuscripts published in English, and searches of references cited in selected articles to identify additional relevant papers. Abstracts sponsored by various societies including the American Urological Association (AUA), European Association of Urology (EAU), and European Society for Medical Oncology (ESMO) were also searched. Background: Bladder cancer is the sixth most common cancer in the United States, and one of the most expensive in terms of cancer care. The overwhelming majority are urothelial carcinomas, more often non-muscle invasive rather than muscle-invasive. Bladder cancer is usually diagnosed after work up for hematuria. While the workup for gross hematuria remains CT urography and cystoscopy, the workup for microscopic hematuria was recently updated in 2020 by the American Urologic Association with a more risk-based approach. Bladder cancer is confirmed and staged by transurethral resection of bladder tumor. One of the main goals in staging is determining the presence or absence of muscle invasion by tumor which has wide implications in regards to management and prognosis. CT urography is the main imaging technique in the workup of bladder cancer. There is growing interest in advanced imaging techniques such as multiparametric MRI for local staging, as well as standardized imaging and reporting system with the recently created Vesicle Imaging Reporting and Data System (VI-RADS). Therapies for bladder cancer are rapidly evolving with immune checkpoint inhibitors, particularly programmed death ligand 1 (PD-L1) and programmed cell death protein 1 (PD-1) inhibitors, as well as another class of immunotherapy called an antibody-drug conjugate which consists of a cytotoxic drug conjugated to monoclonal antibodies against a specific target. Conclusion: Bladder cancer is a complex disease, and its management is evolving. Advances in therapy, understanding of the disease, and advanced imaging have ushered in a period of rapid change in the care of bladder cancer patients.

## 1. Introduction

Bladder cancer is the sixth most common cancer with an estimated 81,400 new cases and 4.5% of all new cancer cases in the US in 2020 [1]. In terms of costs of cancer care, it is estimated to be one of the most expensive cancers prevalent in the United States [2].

In the last few decades, there has been a significant decrease in the bladder cancer-related mortality rate, potentially related to decreased smoking prevalence, improvements in diagnosing bladder cancer, and advances in treatment [3]. Improved understanding of the disease and novel therapies have ushered in a period of rapid change in the field. In this review, we discuss the pathology, diagnosis, staging, radiologic imaging, and management of bladder cancer, highlighting recent developments and research.

## 2. Pathology and Risk Factors

Urothelial carcinoma is the most common bladder cancer, occurring in 90% of cases. Urothelial carcinomas have a propensity for divergent differentiation resulting in a variety of histologic variants which are associated with high-grade and locally advanced disease [4,5]. In one study, urothelial carcinoma with mixed histology was seen in 25% of transurethral resection of bladder tumor (TURBT) specimens [6]. Micropapillary, plastacytoid, and sarcomatoid variants are at higher risk of progressing to muscle-invasive disease, compelling more aggressive treatment to be considered [7].

Pure squamous cell carcinoma is the next most common subtype representing 6-8%, although rates can be up to 50% in regions where schistsomiasis is endemic [8,9]. Pure adenocarcinomas are rare, representing less than 2% of bladder tumors, of which 2/3rd are non-urachal, and 1/3rd are urachal in origin [9].

Bladder cancer is more common in males with median age at diagnosis of 73. Risk factors include smoking which causes approximately half of bladder cancers. Other risk factors include environmental and occupational exposures such as arsenic in drinking water and analine dyes, pelvic radiation, alkylating agents such as cyclophosphamide, chronic bladder irritation/infections, congenital bladder abnormalities including urachal remnants and exstrophy, and gene mutations such as retinoblastoma (RB1) gene mutations, Cowden disease, and Lynch syndrome [2].

## 3. Diagnosis

Bladder cancer is usually diagnosed after workup for hematuria. Patients with gross hematuria should undergo CT urography and cystoscopy. In 2020, the American Urologic Association (AUA) updated their guidelines for microscopic hematuria from the 2012 guidelines, providing a new individualized risk-stratified approach based on age, smoking history, and quality and quantity of hematuria. The 2012 AUA guidelines recommended CT urography and cystoscopy for all patients over 35 years of age with microscopic hematuria. In the 2020 guidelines, low risk patients with microscopic hematuria should have shared decision making for either repeat urinalysis in 6 months or cystoscopy with renal ultrasound. For intermediate risk patients, cystoscopy and renal ultrasound is recommended. For high-risk patients, cystoscopy and CT urogram are recommended [10]. Urine cytology or urine-based tumor markers should not be used in the initial evaluation of microscopic hematuria, but may be used in cases of persistent microscopic hematuria with negative workup and irritative voiding symptoms or risk factors for carcinoma in-situ [10]. Urine cytology may be considered on initial workup when there is suspicion of bladder cancer according to NCCN guidelines [7]. Bladder cancer is confirmed by direct visualization with cystoscopy and transurethral resection of bladder tumor (TURBT).

## 4. Staging

**Figure 1:**
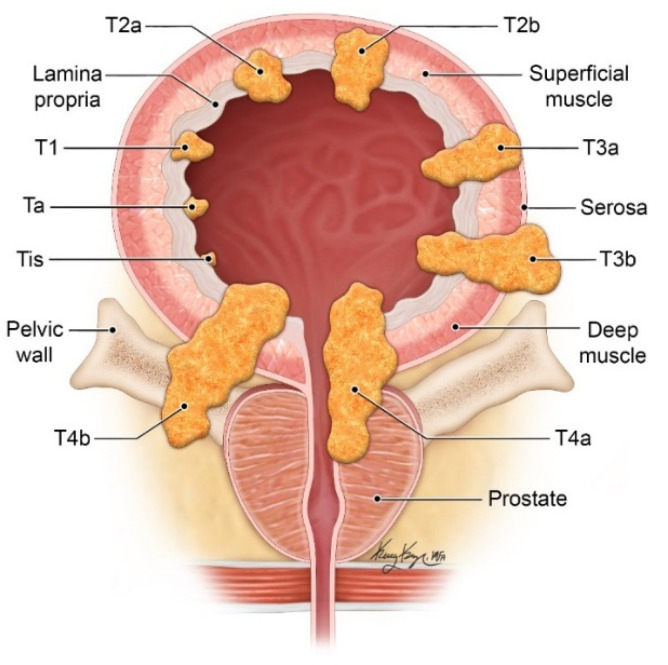
Diagram illustrating the different T-stages of bladder cancer. (© 2021 The University of Texas MD Anderson Cancer Center).

Staging of bladder cancer requires a detailed understanding of bladder anatomy (Figure 1). The urinary bladder is comprised of four layers, from inner to outer: (1) transitional epithelium, (2) submucosa/lamina propria, (3) muscularis, (4) serosa/adventitia. The transitional epithelium, also known as urothelium, is the innermost layer that lines the urinary tract in the renal pelvis, ureters, bladder, and proximal urethra. The second layer is the submucosa/lamina propria, which contains blood vessels and nerves. The third layer is the muscularis layer which is made up of a network of smooth muscle, also called the detrusor muscle. The outer layer of the superior bladder (bladder dome) is covered by serosa which is part of the visceral peritoneum. The remaining areas without serosa have an outer layer of adventitia which is comprised of connective tissue. Beyond the outer layer is the perivesicular fat.

The primary lymphatic drainage for bladder cancer includes the internal iliac, external iliac, obturator, and presacral nodes; and secondarily the common iliac, para-aortic, aortocaval, and paracaval nodes. It is rare for bladder cancer to skip primary nodal drainage sites to metastasize to secondary nodes [11,12].

Bladder cancer is commonly staged using the American Joint Committee on Cancer (AJCC) TNM staging system (Table 1). T describes the primary tumor, N describes lymph node metastases, and M describes distant metastases. Bladder cancer staging carries prognostic significance. The 5-year relative survival rate for carcinoma in situ is 96%, for localized disease is 70%, regional disease is 36%, and distant disease is 5% [13].

## 5. Role of Imaging in Staging:

### 5.1. Computed Tomography (CT)

**Figure 2:**
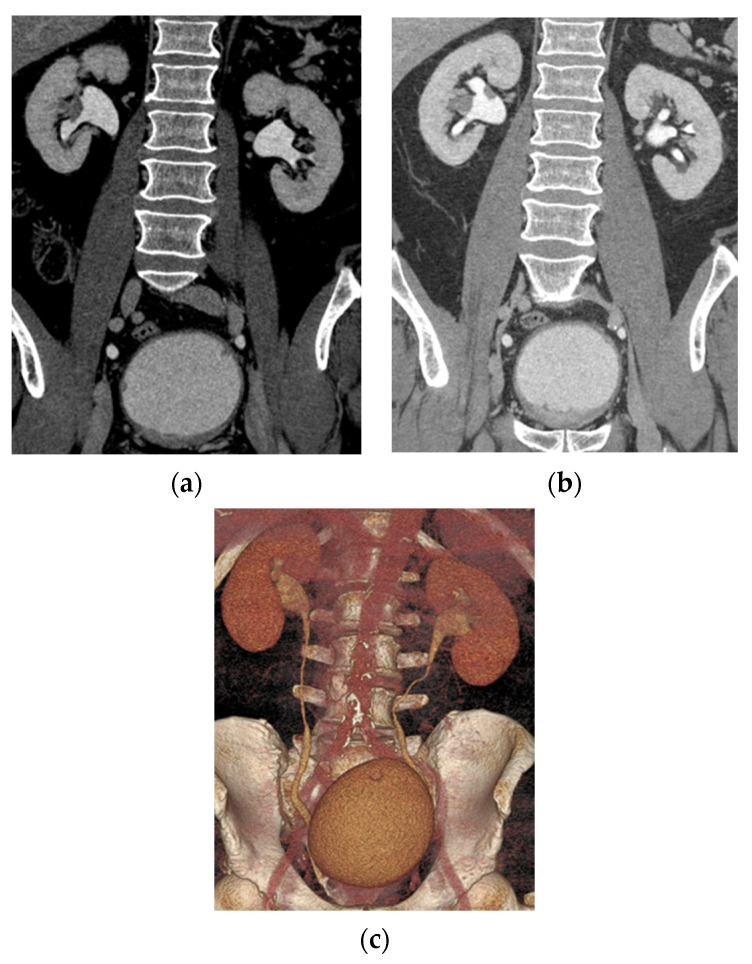
CT urogram showing synchronous upper and lower tract urothelial carcinoma. (**a**) Coronal CT image in the excretory phase shows multiple small bladder lesions (solid white arrow) and synchronous upper tract disease (dashed white arrow) (**b**) Coronal CT image slightly posteriorly, showing multiple upper tract lesions. (**c**) Coronal 3D volume rendering redemonstrates the findings with another bladder lesion (black arrow).

According to NCCN guidelines for patients with suspicion of bladder cancer, CT abdomen and pelvis without and with contrast with excretory phase (CT urography) is recommended prior to TURBT to evaluate the bladder, lymph nodes, potential metastases, and any concurrent upper tract disease (Figure 2) [7]. During primary evaluation and surgical treatment, imaging of the upper tract is recommended if not previously done [7]. At presentation, 2% of patients with bladder urothelial carcinoma will have a synchronous upper tract tumor, and 6% will develop a metachronous lesion [14]. For CT urography, two common techniques are the single-bolus technique and split bolus technique. Single bolus technique involves a single full-dose injection of contrast, followed by scanning the patient during the desired phase of renal parenchymal enhancement, and scanning again during excretory phase. Each phase of contrast is a separate scan which increases radiation dose with the benefit being improved image quality from a larger volume single injection. Split bolus technique involves giving a smaller dose of contrast initially and then the remaining contrast injected after a delay to combine the parenchymal enhancement and excretory phase into a single scan. The decreased number of scans results in a lower radiation dose, although the smaller volume injections may result in less distension of the collecting system and suboptimal parenchymal enhancement [15]. Various additional methods are used to assist in improving distension of the upper tract, including oral or IV hydration or furosemide.

CT urography has a sensitivity of 67–100% and specificity of 93–99% for detection of upper tract urothelial carcinoma [16]. For the detection of bladder cancer, CT urography has a sensitivity of 79–93% and specificity of 83–99% [17,18,19]. For local staging of bladder cancer, CT is better for T3b and T4 disease [19]. Invasion beyond the serosa is seen as nodularity and stranding along the bladder surface, although inflammation and desmoplastic reaction can appear similar, especially after biopsy [20]. Cystoscopy is the considered the gold standard for evaluating the bladder. One study evaluating CT urography in detecting bladder cancer in patients with hematuria or surveillance showed 13/710 false negatives and 47/710 false positives [18]. Of false negatives, 11/13 were limitations of technique as the lesions were unable to be visualized in retrospect, cystoscopy showing carcinoma in situ or urothelial erythema. Two false negatives were related to technical factors, one case related to large post-void residual with suboptimal opacification of the bladder, and one case related to bilateral hip arthroplasties with artifacts obscuring the bladder. All false positive cases were related to errors of interpretation, 12 related to BPH, 9 related to bladder trabeculations, 8 related to post-treatment changes, 3 related to blood clots, 3 related to mistaking normal anatomy for pathology, and 5 without correlate on cystoscopy.

**Figure 3:**
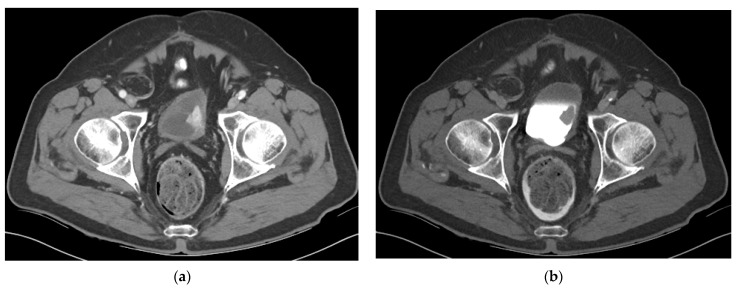
CT of a bladder mass during the urothelial phase and excretory phase (**a**) Axial CT image obtained during the urothelial phase shows a hyperenhancing bladder mass. Bladder tumors tend to be hypervascular and scanning the pelvis during the urothelial phase on CT may aid in tumor evaluation. (**b**) Axial CT image obtained during the excretory phase shows the same mass as a filling defect surrounded by excreted urine in the bladder.

**Figure 4:**
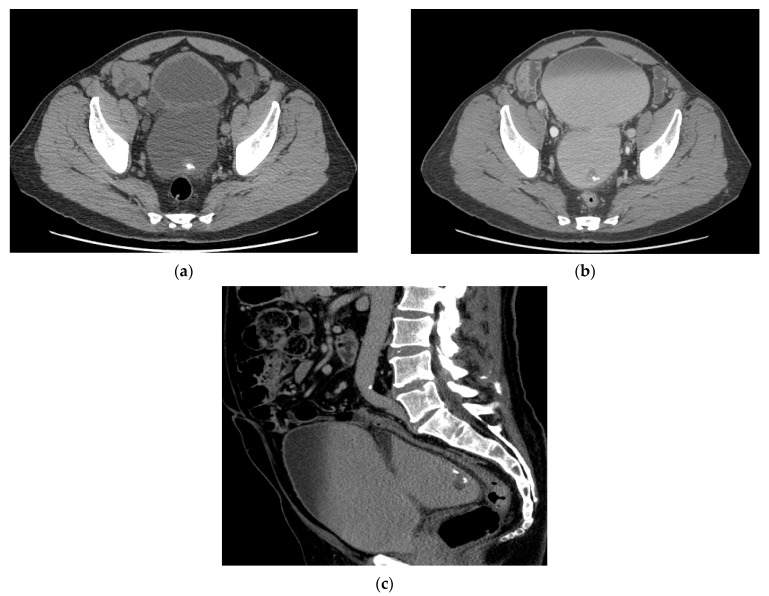
CT urogram with partially calcified urothelial carcinoma within a large bladder diverticulum (**a**) Axial CT image obtained without contrast shows a large bladder diverticulum containing a partially calcified lesion along the posterior aspect representing urothelial carcinoma. (**b**) Axial CT image obtained during the excretory phase and (**c**) Sagittal excretory phase image show the same mass as a filling defect surrounded by excreted urine in the bladder.

On CT, urothelial carcinoma may appear as an intraluminal papillary or nodular mass or focal thickening. Urothelial carcinoma shows early enhancement (Figure 3) and obtaining additional scans of the bladder during the urothelial phase (60 s after contrast injection) may aid in tumor detection. One prospective study of 61 lower tract tumors showed higher sensitivity in bladder tumor detection during the urothelial phase compared to the delayed excretory phase with sensitivity of 89.3% vs. 70.5%, respectively [21]. Calcification is seen in 5% of cases (Figure 4). Small flat lesions may be difficult to visualize and missed. As discussed above, nodularity and stranding outside the bladder can be seen with extraserosal spread of tumor but can also be seen with inflammation and desmoplastic reaction, especially after biopsy (10).

The most common method in determining nodal involvement by CT is size which is not reliable since small nodes may be metastatic and large nodes may be reactive. CT performance for lymph node staging showed a pooled sensitivity of 38% and specificity of 92% [22].

### 5.2. Magnetic Resonance Imaging (MRI)

There is growing interest in multi-parametric MRI for local staging of bladder cancer due to the superior soft tissue contrast of MRI. Multi-parametric MRI is a combination of anatomic T1 weighted-imaging (T1WI) and T2 weighted imaging (T2WI), as well as functional sequences (diffusion weighted imaging and dynamic post-contrast imaging).

**Figure 5:**
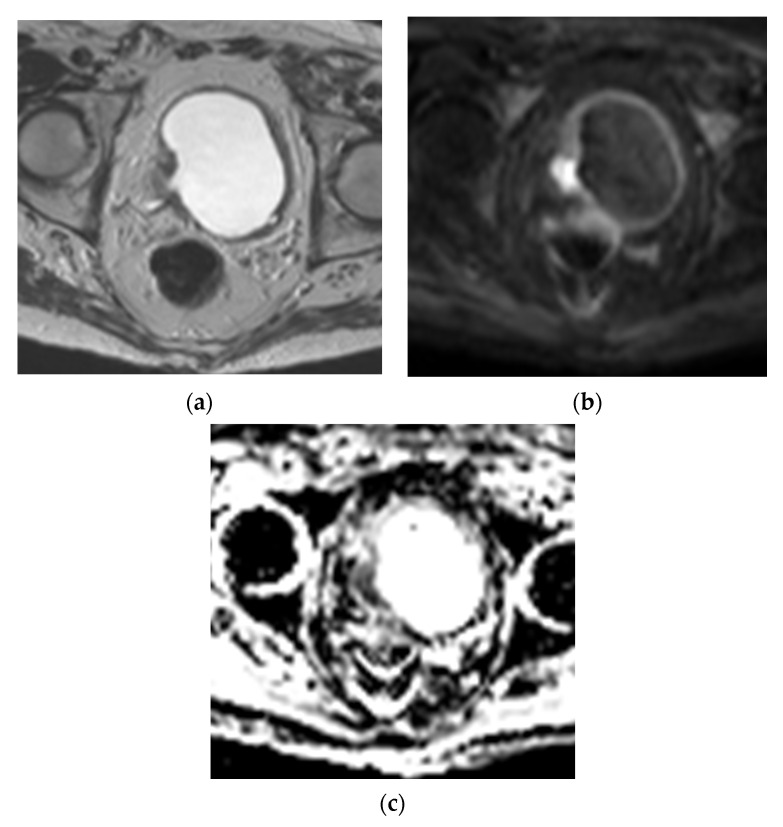
Muscle invasive bladder cancer on MRI (**a**) Axial T2WI shows a muscle invasive mass in the right bladder wall, seen as a T2 intermediate signal lesion (solid white arrow) invasive into the muscularis propria which is T2 hypointense. The mass is located at the ureterovesicular junction, causing hydroureter (dashed white arrow). (**b**) Axial DWI image, showing the hyperintense mass invading the muscularis propria, which is T2 intermediate signal on DWI. (**c**) ADC map showing hypointensity in the area of DWI hyperintensity, confirming restricted diffusion.

**Figure 6:**
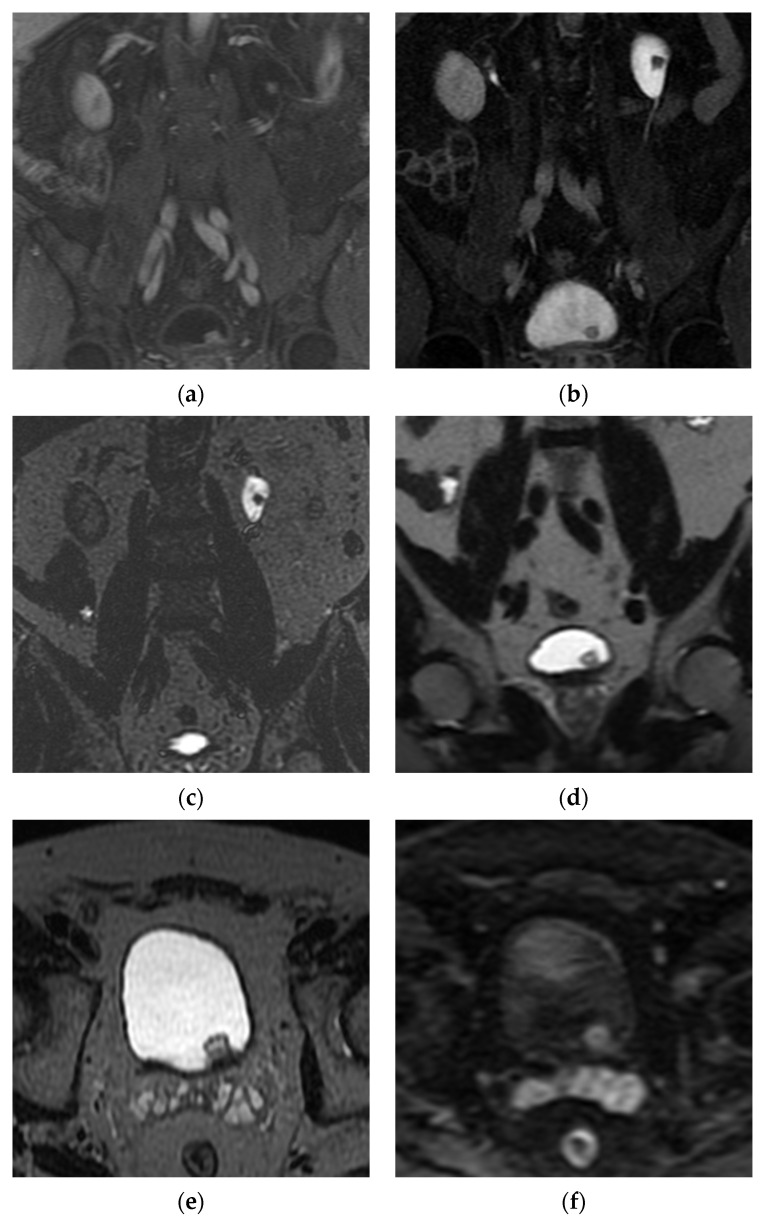
MR urography obtained in a patient with Lynch syndrome showing synchronous upper and lower tract lesions. Pathology of the bladder lesion confirmed non-invasive urothelial carcinoma and upper tract washings were concerning for low grade urothelial carcinoma. (**a**) Coronal post-contrast T1WI shows enhancing lesions in the bladder (solid white arrow) and left renal pelvis (dashed white arrow). (**b**) Coronal post-contrast excretory phase image shows the lesions as filling defects, surrounded by excreted contrast. (**c**) Coronal T2WI shows the upper tract lesion surrounded by T2 hyperintense urine. (**d**) Coronal T2WI of the bladder shows the bladder lesion as T2 intermediate in signal without invasion into the T2 hypointense muscularis propria. (**e**) Axial T2WI of the bladder shows the mass to be non-invasive. (**f**) Axial DWI shows restricted diffusion of the mass.

On MRI, the muscularis layer appears hypointense on T2WI, and intermediate signal on DWI and ADC. The inner layer (urothelium and lamina propria) is not seen on T2WI or DWI/ADC, and shows early enhancement, in contrast to the muscularis which shows late and progressive enhancement. A suspicious lesion is T2-intermediate in signal compared to urine and muscle, shows early enhancement, and restricted diffusion [23]. Examples of muscle-invasive and non-muscle invasive bladder cancer can be seen on Figure 5 and Figure 6, respectively.

A recent systematic review and meta-analysis investigated the diagnostic accuracy of multi-parametric MRI (mpMRI) for local staging [24]. The study showed mpMRI had high pooled sensitivity of 92% and specificity of 88% for differentiating ≤T1 and ≥T2 tumors. For differentiating ≤T2 and ≥T3 tumors, pooled sensitivity (0.71) and specificity (0.77) was suboptimal. Another meta-analysis found a pooled sensitivity and specificity of 0.87 and 0.79 for differentiating ≤T1 and ≥T2, 0.83 and 0.87 for differentiating ≤T2 and ≥T3, and 0.85 and 0.98 for differentiating <T4b and pT4b [25].

In 2018, a panel of experts created the Vesicle Imaging Reporting and Data System (VI-RADS) to standardize imaging and reporting [26]. It consists of a 5-point scale to suggest the risk muscle invasion based on T2WI, DWI, and DCE sequences (Table 2). For T2WI, the reader evaluates lesion size, growth pattern, morphology, location, in addition to evaluating the degree of interruption of the hypointense muscle by T2 intermediate signal tumor. Definitive muscle invasion can be evaluated with DCE and DWI. For DCE, the reader scores the degree of extension of the early enhancing tumor into the non-early enhancing muscle. For DWI, the reader scores the degree of extension of DWI hyperintense tumor into the DWI intermediate signal muscle. The individual scores are used to generate a final 5-point score (Table 2): VI-RADS 1 (muscle invasion highly unlikely), VI-RADS 2 (muscle invasion unlikely to be present), VI-RADS 3 (muscle invasion is equivocal), VI-RADS 4 (muscle invasion likely), VI-RADS 5 (invasion of muscle and beyond the bladder is very likely). A recent overview of VI-RADS summarizes the current literature, showing promising results with inter-reader agreements of 0.73-0.92, sensitivity of 76-95%, and specificity of 77–91% [23].

Another potential benefit of multi-parametric MRI is the evaluation of treatment response. T2WI may play a limited role in post-treatment evaluation due to the difficulty in differentiating bladder cancer from post-treatment inflammation and fibrosis. T2WI for predicting complete pathological response has a sensitivity of 43%, specificity of 45%, and accuracy of 44%. In another study of T2-T4aN0M0 bladder cancer after induction chemotherapy, DWI showed greater specificity and accuracy in predicting pathologic response (sensitivity, specificity, accuracy of 57%, 92%, 80%) compared to T2W (43%, 45%, 44%) and DCE (57%, 18%, 33%), all showing poor sensitivity in detecting residual disease [26,27]. Changes in ADC values after neoadjuvant chemotherapy or chemoradiation have been reported as early indicators of pathologic response [28]. Recent preliminary findings of the PURE-01 study evaluating neoadjuvant Pembrolizumab in muscle-invasive bladder cancer, also evaluated multiparametric MRI as a potential tool in assessing complete tumor response to Pembrolizumab [28]. The study found acceptable interobserver variability (κ 0.5–0.76) and that each parameter (presence or absence of residual disease on T1WI and T2WI, high SI on DWI, and pathological contrast enhancement) was significantly associated with pT0. Notably, no association was found between quantitative mean ADC values after therapy and pathologic response, and qualitative assessment of DWI hyperintensity was pursued. One suggested contributing factor may be the greater histologic complexity in post-immunotherapy stromal tissue with significant T-effector cell recruitment surrounding residual disease, compared to post-chemotherapy tissue [28].

MR urography is used to image the upper tracts and is recommended by multiple guidelines as an alternative to CT urography when necessary such as the AUA, European Association of Urology (EAU), and NCCN [29]. One small prospective study of 20 patients showed equal diagnostic performance between 3T MR urography and triple phase CT urography obtained to exclude upper tract malignancy [29]. Visualization scores noted CT urography to have slightly better visualization of the intrarenal cavity and poorer visualization of the distal ureters, and MR urography showing slightly better visualization of the ureters. One retrospective study between 1.5T MR urography and split-bolus CT urography showed better visibility and diagnostic confidence with CT urography [30]. The limitations of MR urography over CT urography including longer scan time, decreased special resolution, and limited ability to visualize calcifications.

Recently, a consensus conference of the French Society of Genitourinary Imaging was arranged to achieve consensus on imaging protocols including MR urography due to a lack of technical guidelines for imaging protocols [31]. MR urography has two methods of evaluating the collecting system, heavily T2-weighted images similar to magnetic resonance cholangiopancreatography (MRCP), and post-contrast excretory phase imaging. The panel reached consensus that post-contrast excretory phase images were mandatory, but did not reach consensus on whether MRCP-like images should be obtained. Consensus was also reached for other techniques including mandatory DWI when urothelial cancer is suspected. The panel agreed that CT urography remain the first-line examination of the urinary tract, and MR urography used as an alternative in cases of contraindication to CT or radiation concerns.

### 5.3. Positron Emission Tomography (PET)

**Figure 7:**
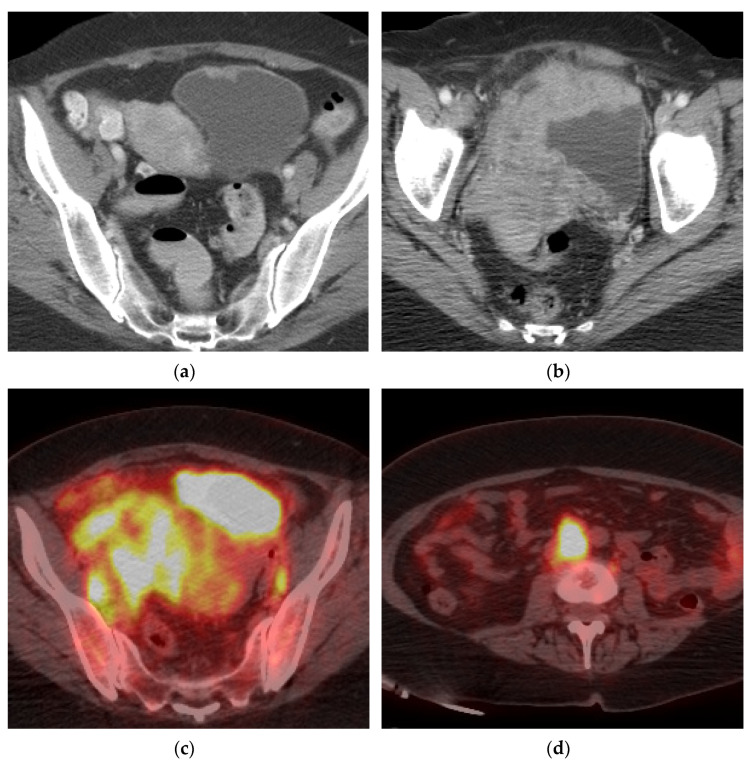

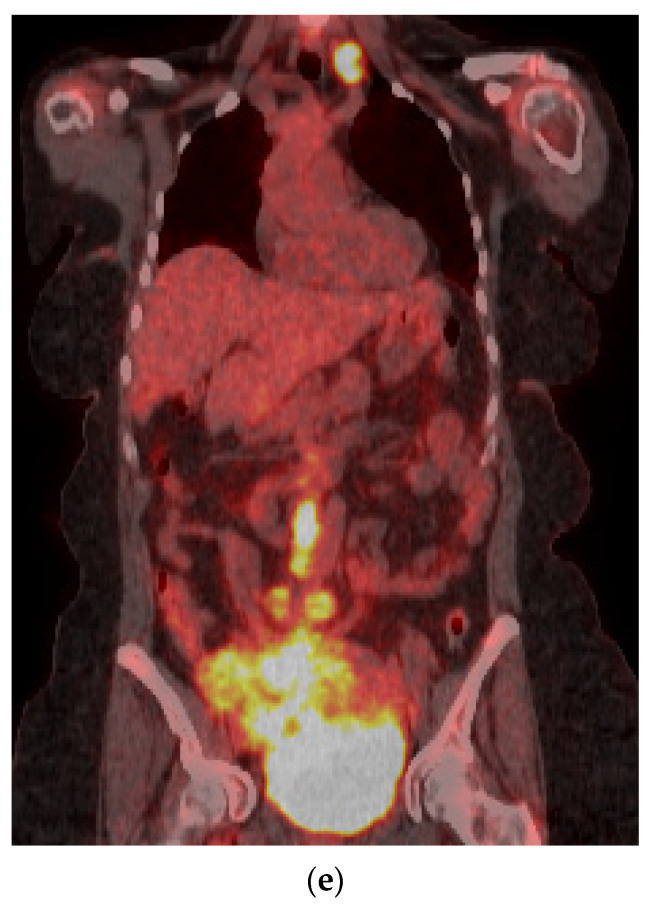
Marked progression of locally advanced bladder cancer and metastatic disease. (**a**) Axial CT image demonstrates a small lesion in the anterior bladder which went untreated for 16 months. (**b**) Follow up axial CT image showing significant progression of the mass which now extends beyond the bladder to involve the uterus, right adnexa, and right pelvic side wall. (**c**) Axial PET/CT image redemonstrating the locally advanced mass with bilateral pelvic lymphadenopathy. (**d**) Axial PET/CT image shows metastatic retroperitoneal lymphadenopathy. (**e**) Coronal PET/CT image also showing metastatic left supraclavicular lymphadenopathy. Biopsy of the left supraclavicular node showed poorly differentiated carcinoma compatible with bladder primary.

FDG PET/CT is of limited use in evaluating the urinary collecting system due to urinary excretion of FDG, but useful in evaluating for distant metastases (Figure 7) [32]. One systematic review and meta-analysis found a pooled sensitivity of 0.82 and pooled specificity of 0.89 for metastatic lesions [33]. FDG PET/CT changed management in 18–68% of patients compared to conventional CT [22]. A recent consensus statement by the European Association of Urology (EAU) and the European Society for Medical Oncology (ESMO) states that FDG PET/CT should be included in the staging of oligometastatic disease when considering radical treatment to minimize the risk of overtreatment [34]. NCCN also suggests FDG PET/CT may be beneficial in select patients with T2 and may change management in ≥cT3 disease [35]. The benefit of FDG PET/CT in lymph node staging is limited. One meta-analysis showed a pooled sensitivity of 57% and specificity of 92% for initial pelvic lymph node staging. In a prospective study of 61 patients undergoing FDG PET/CT prior to radical cystectomy and extended pelvic lymph node dissection, FDG PET/CT did not improve diagnostic accuracy for detecting lymph node metastases compared to conventional CT [36].

Experimental tracers are being investigated, although have not shown sufficient improvement to justify implementation into clinical practice [22]. ^11^C-choline, an analogue of choline which is essential in cell membranes, has increased uptake in malignant tumors as well as the benefit of lack of urinary excretion. One small prospective study found no significant benefit of ^11^C-choline PET/CT over 18F-FDG PET/CT for staging of urothelial carcinoma [37].

FDG PET/MRI is a potential novel approach to the imaging of bladder cancer that leverages the strengths of each—superior contrast resolution and multiparametric assessment with MRI, and metabolic assessment with PET. In one prospective pilot study of 22 PET/MRI exams, PET/MRI had greater accuracy compared to MRI alone for detecting bladder tumor (86% vs. 77%), metastatic pelvic lymph nodes (95% vs. 76%), and non-nodal pelvic malignancy (100% vs. 91%). PET changed suspicion for bladder tumor in 36% (50% increased suspicion, 50% decreased suspicion), for pelvic lymph nodes in 52% (36% increased suspicion, 64% decreased suspicion), and non-nodal pelvis in 9% (100% increased suspicion) [38]. Another recent prospective pilot study of 18 PET/MRI exams showed similar performance of PET/MRI (sensitivity 0.80, specificity 0.56) compared to CT (sensitivity 0.91, specificity 0.43) in the detection of primary bladder tumor. Evaluation of nodal status was limited by the lack of patients with true pathologic lymph nodes [35].

### 5.4. Ultrasound

**Figure 8:**
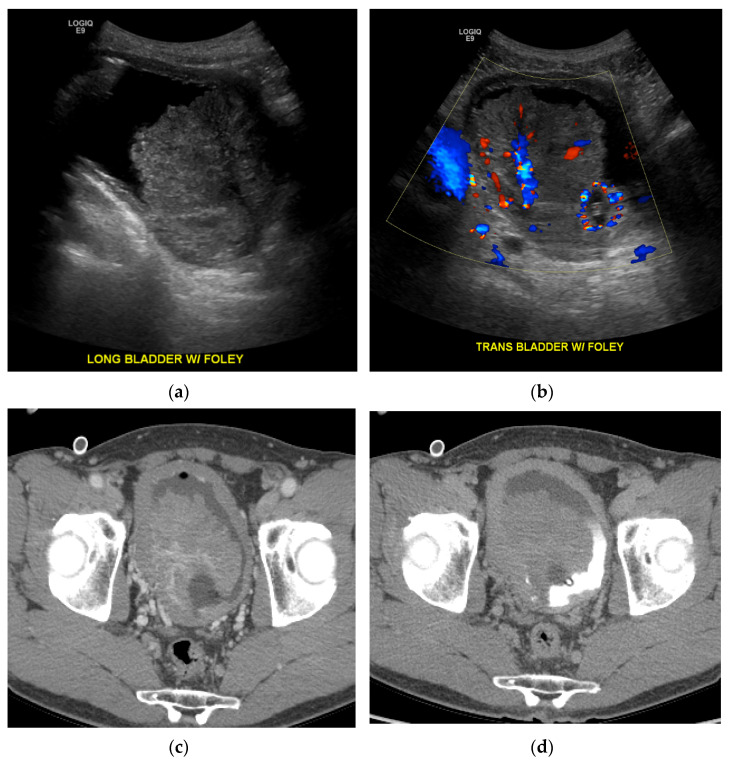
Ultrasound and CT images of non-muscle invasive bladder cancer. (**a**) Longitudinal grayscale ultrasound image of the bladder shows a large mass within the bladder. (**b**) Transverse color Doppler image shows vascularity within the mass. (**c**) Axial portal venous phase CT image shows a central area of enhancement, similar to the color Doppler ultrasound image. (**d**) Axial delayed phase CT image shows the mass partially surrounded by excreted contrast in the bladder.

Ultrasound is not routinely used for bladder cancer staging with transabdominal grayscale US potentially overstaging superficial tumors in 48–49%, and understaging invasive tumors in 5–11% [39]. Ultrasound can be useful for the evaluation of hematuria. In one study of 1007 patients with gross hematuria, ultrasound had a sensitivity of 63% and specificity of 99% in detecting bladder urothelial carcinoma [40]. Bladder carcinoma usually appears as a papillary hypoechoic mass, or an area of focal wall thickening [9]. Tumor can be distinguished from clot by detecting color Doppler flow within the tumor (Figure 8), or mobility of clot during real time examination. On contrast-enhanced ultrasound, bladder cancer is avidly enhancing in contrast to the hypoenhancing muscularis propria. Contrast enhanced ultrasound can better distinguish muscle invasive from non-muscle invasive disease compared to grayscale ultrasound. In one study of 34 patients who underwent both grayscale and contrast-enhanced ultrasound prior to TURBT, contrast-enhanced ultrasound performance approached that of the reference standard of TURBT (area under receiver operating characteristic curve, 0.996) compared to grayscale ultrasound (area under curve, 0.613) [41].

### 5.5. Fluoroscopic/Radiographic Techniques

**Figure 9:**
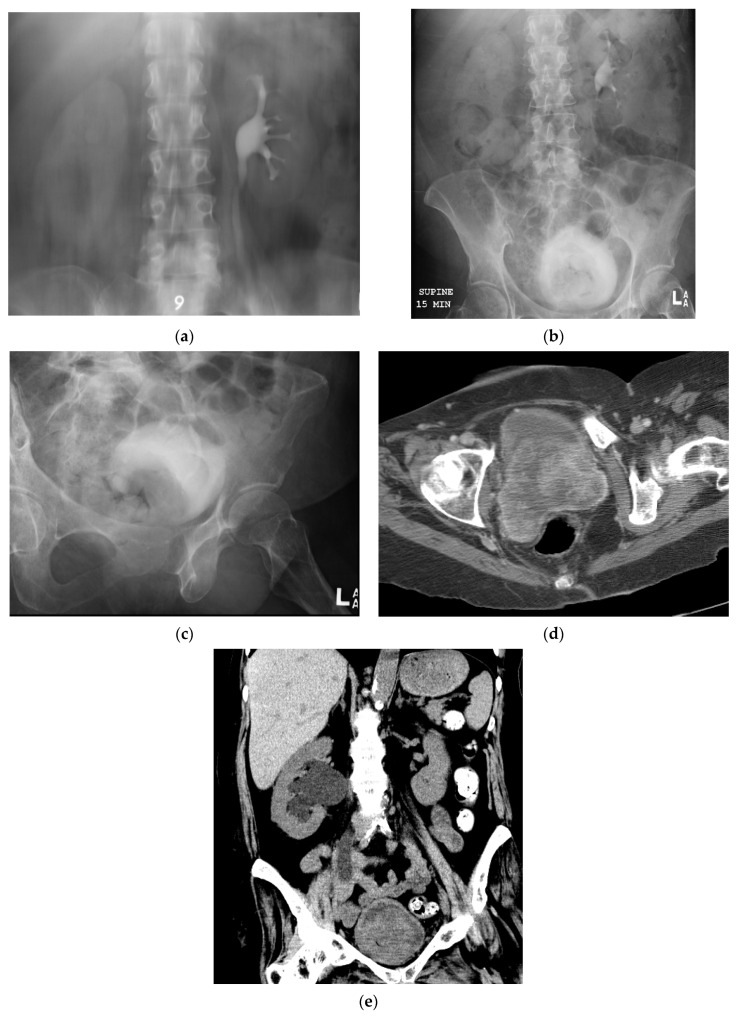
IVP and CT images of locally advanced bladder cancer. (**a**) IVP image of the upper urinary tract at 9-min is without filling defect in the left collecting system to suggest left-sided upper tract disease. The right collecting system is poorly opacified secondary to hydroureteronephrosis, better depicted on CT. (**b**) IVP image at 15 min shows a large filling defect in the bladder. (**c**) Magnified oblique view of the bladder better show a posterior filling defect. (**d**) Axial portal venous phase CT image of the pelvis shows the corresponding mass in the posterior bladder which extends posteriorly to involve the vagina. (**e**) Coronal portal venous phase CT image shows the bladder mass causing right hydrouteronephrosis.

Intravenous urography, also known as intravenous pyelography (IVP), was developed in the 1920′s and historically was the primary imaging method of evaluating the urinary tract [16,42]. Traditionally, excretory urography was used to evaluate for synchronous upper tract disease in patients with bladder cancer [19]. Sensitivity for detecting upper tract lesions ranges from 50–75% [43]. In one study comparing the accuracy of CT urography and excretory urography in detecting and localizing upper tract urothelial carcinoma, CT urography was found to be more accurate with a per-patient sensitivity of 93.5%, specificity of 94.8%, and accuracy of 94.2%. Excretory urography had a per-patient sensitivity of 80.4%, specificity of 81%, and accuracy of 80.8% [43]. Bladder neoplasms can be seen as filling defects (Figure 9); the most sensitive sequences for detecting filling defects are during early bladder filling and post-void [42].

Retrograde pyelography is often performed at the time of cystoscopy, and can aid in problem solving for lesions indeterminate and inconclusive for upper tract urothelial carcinoma on CT or MRI [16]. In a study of patients undergoing surveillance after resected upper tract transitional cell carcinoma, retrograde pyelography read in the endoscopy room had a sensitivity of 71.7% and specificity of 84.7% [44].

## 6. Management

Bladder cancer can be categorized into non-muscle invasive and muscle invasive bladder cancer which differ greatly in management, therapeutic goals, and rates of survival and recurrence. In non-muscle invasive disease, the goal is to prevent progression and limit recurrence. Approximately 75% of bladder cancers are non-invasive which generally have good prognosis with 20–30% rate of progression, but frequently recur with recurrence rates of 60–70% [9,45,46]. In muscle-invasive disease, the goal is deciding for bladder preservation or removal, and whether local bladder therapy alone or if systemic therapy is required. Approximately 25% of bladder cancers are muscle invasive [46].

For suspected bladder cancer, initial evaluation includes abdominal imaging prior to bladder tumor resection. CT urography (CT abdomen and pelvis without and with IV contrast excretory phase) is recommended [7]. MR urography may be appropriate in patients with low GFR or iodinated contrast allergy without acute renal failure. Non-contrast MRI utilizing T2 sequences to evaluate the urinary tract may also be performed if contrast is not permitted. Alternative evaluation of the upper tracts may include retrograde ureteropyelography with renal ultrasound or non-contrast CT, and/or ureteroscopy. MRI pelvis without and with IV contrast can be considered for local staging in cases of sessile or high-grade tumors [7].

The initial evaluation of suspected bladder cancer also includes cystoscopy [7]. Blue-light cystoscopy (BLC) and narrow-band imaging (NBI) are newer enhanced cystoscopy techniques which improve lesion detection compared to conventional white-light cystoscopy (WLC). BLC is a photodynamic diagnostic (PDD) technique using agents such as hexaminolevulinate (HAL) or 5-aminolevulinic acid (5-ALA) which are instilled into the bladder and taken up by urinary epithelial cells. These are used in the formation of photoactive intermediate porphyrins which preferentially accumulate in neoplastic cells and will fluorescence after excitation, appearing as bright pink or red under blue light cystoscopy [47]. Multiple multicenter prospective trials have shown BLC improves detection of non-muscle invasive bladder cancer, in one study showing improved detection by BLC using HAL compared to conventional white light cystoscopy of any malignancy by 23%, papillary lesions by at least 12%, and CIS by 43% [48]. In the same study, 25% of patients had lesions detected exclusively with BLC. Narrow band imaging (NBI) filters white light into two bands of light, blue and green, which are strongly absorbed by hemoglobin and vascular structures [49]. Bladder tumors are better visualized due to their increased vascularity, appearing as dark brown or green against a normal mucosal white or pink background [49]. NBI detects 9–56% more tumors compared to WLC [50]. A meta-analysis of randomized control trials showed no difference in recurrence rates between NBI and HAL-based BLC, lower recurrence with 5-ALA based BLC compared to NBI and HAL-based BLC, and lower recurrence of any 5-ALA and HAL-based BLC and NBI compared to WLC [51]. Another meta-analysis found similar sensitivity and specificity between HAL and 5-ALA based BLC [52].

TURBT is performed to confirm pathology and to determine depth of invasion with the goal of resecting all visible tumor. A sample is considered adequate if it contains muscle to allow for determination of muscle invasion [7]. Further management will depend on whether non-muscle invasive or muscle-invasive disease is present.

For non-muscle invasive bladder cancer, repeat (restaging) TURBT is often recommended because of the chance of upstaging disease with a change in management in 24–49% in high-grade T1 tumors [46]. Residual tumor after initial TURBT is common [53], and recurrence rates are lower in patients who have undergone repeat TURBT, 16% versus 58% in patients without repeat TURBT [53,54]. Depending on risk factors for recurrence and progression such as high-grade disease, higher T-stage (Ta vs. Tis or T1), large size, history of frequent recurrences, multifocality, histologic variants, lymphovascular invasion, and greater depth of invasion (deep T1 tumor), management may include induction with maintenance intravesical chemotherapy, as opposed to single dose post-TURBT intravesical chemotherapy in the absence of risk factors [7,55]. Gemcitabine and mitomycin are the most commonly used intravesical chemotherapy agents in the United States. Induction with maintenance intravesical immunotherapy with Bacillus Calmette-Guérin live attenuated vaccine (BCG) is also recommended for high-risk non-muscle invasive bladder cancer, although since 2012, a BCG shortage has existed due to demand surpassing production capacity [56]. A 26% reduction in risk of progression can be seen in carcinoma in situ patients treated with intravesical BCG compared to intravesical chemotherapy. Cystectomy may be considered for the highest risk patients as an alternative to intravesical BCG, including patients ≥ 70 years of age with tumors ≥ 3cm and concomitant carcinoma in-situ [57], and those with deep lamina propria invasion and lymphovascular invasion [58].

For muscle invasive bladder cancer, neoadjuvant cisplatin-based chemotherapy followed by radical cystectomy and lymph node dissection is considered standard treatment. Randomized control trials have shown cisplatin-based neoadjuvant therapy improves overall survival by 5–10% [59]. Neoadjuvant therapies include MVAC (methotrexate, vinblastine, doxorubicin, and cisplatin), ddMVAC (dose dense MVAC), CMV (cisplatin, methotrexate, and vinblastine), and GC (gemcitabine and cisplatin). No established guidelines currently exist as to which neoadjuvant therapy is best. Preliminary data from the phase III GETUG/AFU V05 VESPER trial shows complete pathologic responses and organ confined status were more frequent in dose dense MVAC versus GC with survival results pending [60]. Another large phase III trial showed similar survival between MVAC and GC, with GC showing better safety profile and tolerability [61]. The PURE-01 trial showed 42% complete remission for neoadjuvant immunotherapy with pembrolizumab with the first survival outcomes showing favorable prognosis with exception of ypN+ showing recurrence free survival of 39.3% at 24 months [62]. The NIAGRA trial investigating neoadjuvant durvalumab with GC, and KEYNOTE-866 trial investigating pembrolizumab with GC, are ongoing.

Pelvic lymph node dissection is recommended at the time of radical cystectomy, at minimum to include the bilateral external iliac, internal iliac, and obturator lymph nodes [18,19]. The lymph node dissection is bilateral as contralateral lymph node involvement is common. In one study, contralateral nodes were seen in 41% of patients with unilateral tumor [63]. The recommended superior extent of lymph node dissection (i.e., extended lymph node dissection) is not yet defined [18,19]. Extended lymph dissection includes presacral and common iliac nodes to the aortic bifurcation, and may include retroperitoneal nodes to the level of the inferior mesenteric artery. EAU and ESMO guidelines state the extended lymphadenectomy is usually considered as standard [34,64]. Results of the first randomized phase III trial, LEA AUO AB 25/0, were published in 2019 that investigated extended versus limited pelvic lymph node dissection at the time of radical cystectomy. A trend towards increased recurrence free survival, cancer free survival, and overall survival was observed which did not reach statistical significance. In the extended lymph node dissection arm, 4 patients out of the 44 pN+ patients had skip metastases in the common iliac or presacral nodes, and the patients would have been diagnosed as pN0 had they not undergone extended lymph node dissection [65,66]. Another trial evaluating extended versus standard lymph node dissection, SWOG S1011, is ongoing with a few notable differences from the LEA trial. The SWOG S1011 trial excludes T1 disease and includes patients undergoing neoadjuvant chemotherapy, in contrast to the LEA trial which included T1-T4 disease without any patients receiving neoadjuvant chemotherapy [67].

In women, radical cystectomy involves anterior pelvic exenteration with resection of the bladder, urethra, uterus, ovaries, fallopian tubes, and anterior vaginal wall. In men, radical cystectomy involves resection of the bladder, prostate, and seminal vesicles [68].

Radiotherapy for non-muscle invasive bladder cancer is not generally indicated, but may be considered as a potentially curative alternative for patients with recurrent Ta-T1 tumors following BCG therapy without diffuse Tis who are not candidates for cystectomy. For non-metastatic muscle invasive bladder cancer, multimodal bladder preserving (trimodal) therapy may be considered. Trimodal therapy consists of TURBT, chemoradiation, and cystoscopic re-evaluation. Evidence for concurrent chemoradiation is based on a series of studies including a pooled analysis of Radiation Therapy Oncology Group studies, a large randomized control trial, and large single institutional studies [69,70,71,72]. The ideal candidate for trimodal therapy includes smaller solitary lesions, minimal T2 disease, completely resected tumor after TURBT, and lack of tumor related hydronephrosis [46]. No completed randomized control trials directly compare trimodal therapy to radical cystectomy. Concurrent radiotherapy may also be considered for non-operative patients, or for palliation in patients with metastatic disease. Adjuvant radiation may be considered after cystectomy based on pathologic findings at resection. A recent consensus statement by the European Association of Urology and European Society for Medical Oncology states that radiotherapy for bladder preservation should be performed with Intensity-Modulated Radiation Therapy (IMRT) or image-guided radiation therapy (IGRT) [34]. The panel also reached consensus that radiation dose escalation above standard radical doses to the primary site in cases of bladder preservation by IMRT or brachytherapy is not recommended. The RAIDER trial is currently seeking to evaluate the potential value of dose-escalation [73]. The difficulty with irradiating the bladder beyond standard doses is secondary to bladder mobility which can markedly change in shape and volume. Utilizing advances in image guidance, the study incorporates the use of cone-beam CT to create a library of plans to cover the range of bladder positions and create a “plan of the day” for better tumor targeting and minimizing non-target toxicity. Radiotherapy utilization for muscle-invasive disease has been increasing based on SEER data from 1992–2013 [74], but historically underutilized with a 2005 study showing potentially 58% of patients which should have received radiotherapy based on evidenced-based algorithms but utilization ranging from 4–26% [75].

Data on adjuvant chemotherapy is lacking and its role is not yet defined. One important randomized trial showed adjuvant therapy improved 5-year progression free survival although did not change overall survival [76].

Immunotherapy against bladder cancer has been in practice for decades with intravesical BCG, although the exact mechanism BCG induces an anti-tumor response has not been fully elucidated [77]. Newer advances in immunotherapy for bladder cancer have mainly focused on immune checkpoint inhibitors, particularly programmed death ligand 1 (PD-L1), programmed cell death protein 1 (PD-1) inhibitors. FDA approved immune checkpoint inhibitors for bladder cancer include atezolizumab, avelumab, and durvalumab (PD-L1 inhibitors), as well as nivolumab and pembrolizumab (PD-1 inhibitors). Immune checkpoint inhibitors may be suitable against bladder cancer potentially because of similar mechanisms between melanoma, non-small cell lung cancer, and bladder cancer with chronic carcinogenic exposure causing high levels of malignancy associated neo-antigen presentation [77]. Immune checkpoint inhibitors are generally second-line therapy after platinum-based therapy. Atezolizumab and pembrolizumab are approved for first-line use in cisplatin-ineligible patients with tumors expressing PD-L1, or patients unable to receive any platinum based therapy regardless of PD-L1 expression [78]. Approximately 30-50% of patients may be ineligible for cisplatin due to impaired renal function and low performance status, in part related to bladder cancer being a disease of the elderly [1]. Avelumab is also recommended as maintenance therapy in patients who have not progressed after first-line platinum-based therapy [79]. In April 2020, Avelumab was FDA approved based on the JAVELIN Bladder 100 trial showing significantly prolonged overall survival in patients with unresectable locally advanced or metastatic urothelial carcinoma on maintenance avelumab plus best supportive care (71.3% 1-year overall survival), compared to best supportive care alone (58.4% 1-year overall survival) [79].

Enfortumab vedotin is another class of immunotherapy FDA approved for bladder cancer, an antibody-drug conjugate, consisting of antibodies targeting Nectin-4 conjugated with the cytotoxic agent monomethyl auristatin E (MMAE) [80]. Nectin-4 is an antigen highly expressed in urothelial carcinoma [80]. Enfortumab vedotin was recently FDA approved for third line therapy after the EV-201 study investigated Enfortumab vedotin in patients with locally advanced or metastatic disease who were previously treated with platinum therapy and PD-1 or PD-L1 inhibitor. The study showed a 44% objective response rate, including 12% complete responses. The median duration of response was 7.6 months [80].

Metastasis is seen in 5% of bladder cancer patients at the time of diagnosis [1], and up to half of patients that have undergone cystectomy will relapse with distant disease [81]. Systemic therapies involve the same agents used in locally advanced disease.

## 7. Imaging Surveillance

For non-muscle invasive bladder cancer without risk factors, CT surveillance is not recommended [82]. Routine surveillance for upper tract disease in asymptomatic low-risk patients is also not recommended because of the low incidence of less than 1% [82]. In one study of 934 patients, 51 upper tract urothelial carcinomas were diagnosed, 15 of which were diagnosed on routine imaging, the remaining diagnosed when the patients became symptomatic. The imaging efficacy was 0.49% (15 out of 3074 CT exams) [83].

For muscle invasive bladder cancer, NCCN suggests follow up abdominopelvic imaging at 3–6 month intervals for two years, and then annually for up five years, and as indicated afterwards [7]. After radical cystectomy, patients who recur usually present with distant recurrence rather than local recurrence. In one study of 311 patients with recurrence, 75% were distant recurrence with median time to recurrence of 12 months, and 25% were local recurrence with median time to recurrence of 18 months [84]. Older literature reports nearly 40% local recurrence after radical cystectomy with more contemporary literature showing 6-13% local recurrence. Improved local control may be related to improved surgical techniques and neoadjuvant therapy [81]. Up to 50% of patients who undergo cystectomy will have distant recurrence, the most common non-nodal sites being bone, lung, and liver, and less frequently brain, skin, vagina, and peritoneum [81]. Notably with the advances in immunotherapies, the concept of pseudo-progression has been described in melanoma, thought to be related to immune infiltration causing tumors to appear radiologically larger. However, in urothelial cancer, the most common radiologic outcome in single-agent immunotherapy is progression, and a recent EAU-ESMO consensus statement states that pseudo-progression from immunotherapy has not been demonstrated in urothelial cancer [34].

## 8. Summary

Bladder cancer is a complex disease with a wide range of tumor behavior and variable clinical outcome. Imaging plays an essential role in staging and follow-up of bladder cancer. Improvements in understanding of the disease and novel therapies have ushered in a period of rapid change and provided new armamentarium in the fight against bladder cancer and improving patient outcomes.

## Figures and Tables

**Table 1:** AJCC TNM staging (8th edition).

T Stage	Description
Tx	Primary tumor unable to be evaluated
T0	No evidence of primary tumor
Ta	Noninvasive papillary carcinoma
Tis	Carcinoma in situ
T1	Tumor invades lamina propria but does not involve bladder muscle
T2	Tumor invades bladder muscle
T2a	Tumor invades superficial muscle (inner half)
T2b	Tumor invades deep muscle (outer half)
T3	Tumor invades perivesical tissue
T3a	Microscopic perivesical invasion
T3b	Macroscopic perivesical invasion
T4	Tumor invades adjacent organs
T4a	Tumor invades prostate, seminal vesicles, uterus, or vagina
T4b	Tumor invades pelvic wall or abdominal wall
N stage	
Nx	Regional lymph nodes cannot be evaluated
N0	Single regional lymph node metastasis in the true pelvis (hypogastric, obturator, external iliac, or presacral)
N1	2+ regional lymph node metastases in the true pelvis
N3	Lymph node metastasis to common iliac lymph nodes
M stage	
M0	No distant metastasis
M1	Distant metastasis
M1a	Non-regional lymph node metastasis
M1b	Non-lymph node distant metastasis

**Table 2:** VI-RADS scoring system [26].

Score	Description
T2WI Score Structural category (SC)	Muscularis propria is T2 hypointense Tumor is T2 intermediate in signal
SC 1	Uninterrupted low signal intensity (SI) line representing the muscularis propria. Lesion < 1 cm Exophytic tumor +/− stalk +/− thickened inner layer (urothelium and lamina propria)
SC 2	Uninterrupted low signal intensity (SI) line representing the muscularis propria Lesion > 1 cm Exophytic tumor with stalk +/− high SI thickened inner layer, when present. Sessile/broad-based tumor with high SI thickened inner layer, when present
SC 3	Lack of category 2 findings with no clear disruption of low SI muscularis propria. Associated presence of Exophytic tumor without stalk Sessile broad based tumor without high SI thickened inner layer.
SC 4	Intermediate SI tumor interrupts low SI line (muscularis propria)
SC 5	Intermediate SI tumor extends into extravesical fat
DCE Score Contrast-enhanced category (CE)	Tumor and inner layer enhance early Muscle should not enhance early
CE 1	No early enhancement of muscularis propria
CE 2	No early enhancement of muscularis propria with early enhancement of inner layer
CE 3	Lack of category 2 findings with no clear disruption of muscularis propria
CE 4	Early enhancing tumor extends into muscularis propria
CE 5	Early enhancing tumor extends to entire bladder wall and to extravesical fat
DWI/ADC score Diffusion weighted category (DW)	Tumor is hyperintense on DWI, hypointense on ADC Muscularis propria is intermediate SI on DWI Stalk and inner layer are low SI on DWI
DW 1	Intact intermediate SI muscularis propria on DWI Lesion < 1 cm
DW 2	Intact intermediate SI muscularis propria on DWI Lesion > 1 cm
DW 3	Lack of category 2 findings with no clear disruption of muscularis propria
DW 4	Tumor (high SI on DWI/low SI on ADC) extends into muscularis propria
DW 5	Tumor (high SI on DWI/low SI on ADC) extends to entire bladder wall and extravesical fat
Final Score	T2WI helpful especially for VI-RADS 1-3 DWI and DCE are dominant sequences for risk estimate, especially for VI-RADS 4-5
VI-RADS 1	Muscle invasion highly unlikely SC 1, CE 1, and DW 1
VI-RADS 2	Muscle invasion unlikely SC 2 + CE 2 and DW 2 SC 3 + CE 2 and DW 2
VI-RADS 3	Muscle invasion equivocal SC 3 + CE 3 and/or DW 3 (or below)
VI-RADS 4	Muscle invasion likely SC 4 + CE 4 and/or DW 4 (or below) SC 5 + CE 4 and/or DW 4 (or below)
VI-RADS 5	SC 4 + CE 5 and/or DW 5 SC 5 + CE 5 and/or DW 5

## Data Availability

Not applicable.
